# Aflatoxin B_1_ and M_1_ Degradation by Lac2 from *Pleurotus pulmonarius* and Redox Mediators

**DOI:** 10.3390/toxins8090245

**Published:** 2016-08-23

**Authors:** Martina Loi, Francesca Fanelli, Paolo Zucca, Vania C. Liuzzi, Laura Quintieri, Maria T. Cimmarusti, Linda Monaci, Miriam Haidukowski, Antonio F. Logrieco, Enrico Sanjust, Giuseppina Mulè

**Affiliations:** 1 Institute of Sciences of Food Production, National Research Council of Italy (CNR), via Amendola 122/O, Bari 70126, Italy; martina.loi@ispa.cnr.it (M.L.); francesca.fanelli@ispa.cnr.it (F.F.); vania.liuzzi@ispa.cnr.it (V.C.L.); laura.quintieri@ispa.cnr.it (L.Q.); teresa.cimmarusti@ispa.cnr.it (M.T.C.); linda.monaci@ispa.cnr.it (L.M.); miriam.haidukowski@ispa.cnr.it (M.H.); antonio.logrieco@ispa.cnr.it (A.F.L.); 2 Department of Economics, University of Foggia, via Napoli 25, Foggia 71122, Italy; 3 Department of Biomedical Sciences, University of Cagliari, Cittadella Universitaria, Complesso Universitario, SP Monserrato-Sestu Km 0.700, Monserrato 09042, Italy; pzucca@unica.it (P.Z.); sanjust@unica.it (E.S.)

**Keywords:** laccase, *Pleurotus*, mycotoxins, aflatoxin B_1_, aflatoxin M_1_, biodegradation, redox mediators

## Abstract

Laccases (LCs) are multicopper oxidases that find application as versatile biocatalysts for the green bioremediation of environmental pollutants and xenobiotics. In this study we elucidate the degrading activity of Lac2 pure enzyme form *Pleurotus pulmonarius* towards aflatoxin B_1_ (AFB_1_) and M_1_ (AFM_1_). LC enzyme was purified using three chromatographic steps and identified as Lac2 through zymogram and LC-MS/MS. The degradation assays were performed in vitro at 25 °C for 72 h in buffer solution. AFB_1_ degradation by Lac2 direct oxidation was 23%. Toxin degradation was also investigated in the presence of three redox mediators, (2,2′-azino-bis-[3-ethylbenzothiazoline-6-sulfonic acid]) (ABTS) and two naturally-occurring phenols, acetosyringone (AS) and syringaldehyde (SA). The direct effect of the enzyme and the mediated action of Lac2 with redox mediators univocally proved the correlation between Lac2 activity and aflatoxins degradation. The degradation of AFB_1_ was enhanced by the addition of all mediators at 10 mM, with AS being the most effective (90% of degradation). AFM_1_ was completely degraded by Lac2 with all mediators at 10 mM. The novelty of this study relies on the identification of a pure enzyme as capable of degrading AFB_1_ and, for the first time, AFM_1_, and on the evidence that the mechanism of an effective degradation occurs via the mediation of natural phenolic compounds. These results opened new perspective for Lac2 application in the food and feed supply chains as a biotransforming agent of AFB_1_ and AFM_1_.

## 1. Introduction

Laccases (LCs, benzenediol: oxygen oxidoreductase, EC 1.10.3.2) are multicopper oxidases widely distributed in plants, bacteria, insects, and fungi [1]. Among fungi, white rot basidiomycetes, such as *Pleurotus* spp. are the most efficient producers of LCs [2]. Laccases typically contain four cupric ions, classified within three distinct spectroscopic types, T1, T2, and T3 [3]. They are essential for the one-electron oxidation of a reducing substrate and for the reoxidation of the enzyme by means of molecular oxygen, which is in turn reduced to water. Some “white” or “yellow” fungal laccases have been described [4,5], lacking the T1 cupric ion, which confers the blue color to the enzyme.

*Pleurotus pulmonarius* Fr. (Quél.), or Indian oyster mushroom, is an edible mushroom known for its medicinal properties and biotechnological potential [6]. It produces several LC isoforms, which are encoded by complex multi-gene families. LCs have different substrate specificity, catalytic properties, regulatory mechanisms and localization. Their synthesis and secretion depends upon nutrient levels, culture conditions, developmental stage, and can be increased by the addition of a wide range of inducers to cultural media [7].

LC catalyzes the oxidation of phenols, aromatic amines, and other non-phenolic compounds, while reducing molecular oxygen to water; LC activity can be further extended to non-phenolic substrates by the use of synthetic or natural redox mediators [8]. The mediators, after being oxidized by LC, diffuse out of the active site and oxidize recalcitrant compounds which possess high redox potential or high molecular weight. Being structurally diverse, different mediators may act on chemically-unrelated compounds, widening LC substrate range [9].

Several compounds have been used as redox mediators in the laccase mediator system (LMS). Synthetic mediators such as 2,2-azino-bis-[3-ethylbenzo-thiazolin-sulfonate] (ABTS), 2,2,6,6-tetramethylpiperidine-*N*-oxyl (TEMPO), and 1-hydroxybenzotriazole (HBT), have been widely used in many biocatalytic processes [10,11]. However, their use is limited due to their high cost, toxicity, and the high mediator-substrate molar ratio needed.

Being a green catalyst with a broad range of substrates, LC has been industrially applied since the early 1990s in chemical synthesis, the food industry, and bioremediation [12].

In addition, LC and LC-like activities in crude fungal extracts have been positively correlated with mycotoxin degradation [13,14,15,16], though neither the mechanism of action, nor the degradation products, have been yet fully elucidated.

Mycotoxins are secondary metabolites mainly produced by *Aspergillus*, *Penicillium*, and *Fusarium* spp., which display toxic, carcinogenic, teratogenic, and mutagenic activity towards humans and animals, and contaminate staple food commodities worldwide. Aflatoxins are mycotoxins produced by *Aspergillus* spp., and aflatoxin B_1_ (AFB_1_) is the most toxic: it has been classified by the International Agency for Research on Cancer (IARC) as Group 1, carcinogenic to humans, and it is known for its teratogenic, hepatotoxic, and immunosuppressive effects on humans and animals [17].

Due to their stability, which confers resistance to physical and chemical treatments of food processing, aflatoxins can persist and are usually found in cereal-based and animal products.

Aflatoxin M_1_ (AFM_1_) is the animal catabolic product of AFB_1_ and contaminates milk and dairy products. AFM_1_ is classified in group 2B by the IARC due to its demonstrated hepatotoxic and carcinogenic effect on animals, although its toxicity is one order of magnitude lower than AFB_1_ [17].

Due to aflatoxin contamination, every year billions of dollars are lost along the food and feed supply chain worldwide [18], constituting a huge economic problem and a public health concern. 

In the current study we purified a LC isoform from *P. pulmonarius*, a well characterized LC producer and a source of LC isozymes with recognized biotechnological potential in the field of bioremediation. We tested the degrading activity of purified LC towards AFB_1_ and AFM_1_, elucidating the effect of direct and mediated oxidation using a model synthetic mediator, ABTS, and two naturally-occurring phenols, acetosyringone (AS) and syringaldehyde (SA).

## 2. Results

### 2.1. LC Production and Purification

After 24 days of static incubation, the total activity (enzymatic activity, EU) of the crude extract (4 L) was 17,120 EU with specific activity of 6 U/mg. The purification steps for LC are detailed in Table 1. The apparent increase in total activity after Ca-phosphate gel might be due either to inhibition effect or interference with ABTS analysis by dark brown pigments (probably arising from oxidation/polymerization of ferulic acid; these pigments are nearly totally removed during this first purification step).

As preliminary steps, the batch treatment with calcium phosphate gel and the first anion exchange chromatography on diethylamino ethyl (DEAE) cellulose were performed to remove the majority of the brown pigments and contaminating proteins and resulted in the greatest increase in specific activity, from 6 to 2068 U/mg. In the last two chromatographic steps LC activity was further purified from remaining impurities and from the other contaminant proteins. This strategy resulted in 1820-fold purification with 12% final yield of laccase enzyme with respect to the crude extract.

### 2.2. Zymography

**Figure 1:**
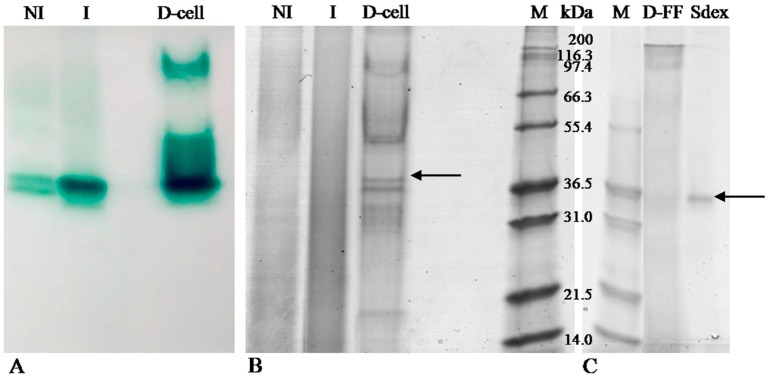
Zymography (**A**) using ABTS as the substrate and SDS PAGE (**B** and **C**) of *P. pulmonarius* LC preparations. NI-not induced; I-induced; D-cell-sample after DEAE cellulose, D-FF-sample after DEAE FF, Sdex-sample after Superdex, M-Marker. The arrows indicate Lac2 bands.

Figure 1, panel A shows the zymogram of extracellular LC activity by using ABTS as the substrate; protein bands exhibiting activity in zymogram were also compared with the related electrophoretic pattern stained with Coomassie stain (Figure 1, panel B and C).

In particular, the not induced (NI) sample putatively produced two laccase isoforms corresponding to a molecular weight ranging from 31 to 36.5 kDa in the electrophoretic profile stained with Coomassie (Figure 1, panel B). The induced (I) sample showed an analogous zymogram pattern with the exception of the band showing a more intense activity than the NI sample due to LC induction by ferulic acid. However, the zymogram was more sensitive than Coomassie staining, as previously reported [19]. LC activity with ABTS was still clearly detectable when as low as 0.3 µg of proteins were loaded on SDS-PAGE (data not shown). After the final purification step with Superdex, Lac2 appeared as one single band in SDS PAGE (Figure 1, panel C).

### 2.3. Laccase Identification by MS/MS

Table 2 summarizes the identification of the band digested from the polyacrylamide gel with the highest score. After protein digestion, the resulting peptide mixture was analyzed in data-dependent MS/MS acquisition mode, The acquired MS data were processed by Proteome Discoverer software (version 1.4, 2012, Thermo Scientific, San José, CA, USA) and searched against a customized DB containing all *Pleurotus* spp. protein sequences present in UniProt. As a result, seven unique peptides were detected and assigned to Lac2 and Lac4 of *P. pulmonarius* and *P. sajor-caju*, respectively. In Table 2 the peptide sequences identified by MS and matching with part of Lac2 and Lac4 sequences in the Uniprot DB are reported. Considering that *P. pulmonarius* and *P. sajor-caju* are synonymous [20], and that the strain used in this study belonged to *P. pulmonarius* species, we identified our protein as Lac2 of *P. pulmonarius*.

### 2.4. In Vitro Degradation of AFB_1_ and AFM_1_ with Laccase and Redox Mediators

**Figure 2:**
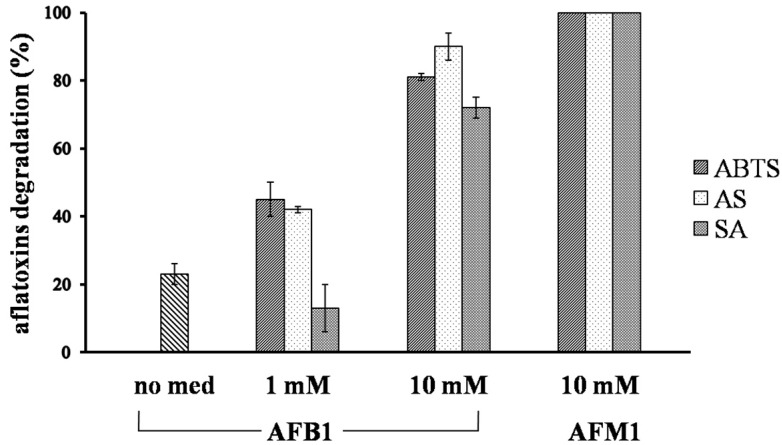
AFB_1_ and AFM_1_ degradation (%) after three days of incubation at 25 °C, performed by Lac2 and the respective redox mediator in buffered solution (1 mM sodium acetate pH 5). ABTS-[2,2′-azino-bis-(3-ethylbenzothiazoline-6-sulfonic acid)]; AS-acetosyringone, SA-syringaldehyde. Values are the mean of three replicates and the error bars represent the standard error measured between independent replicates.

**Figure 3:**
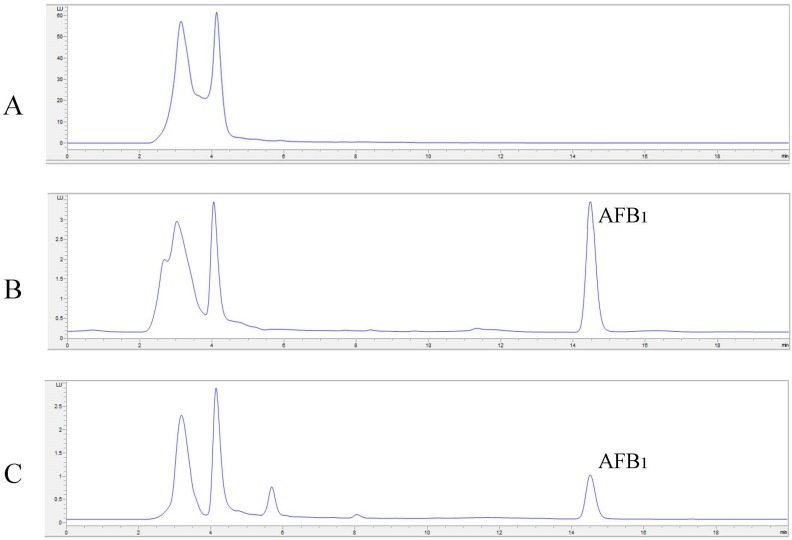

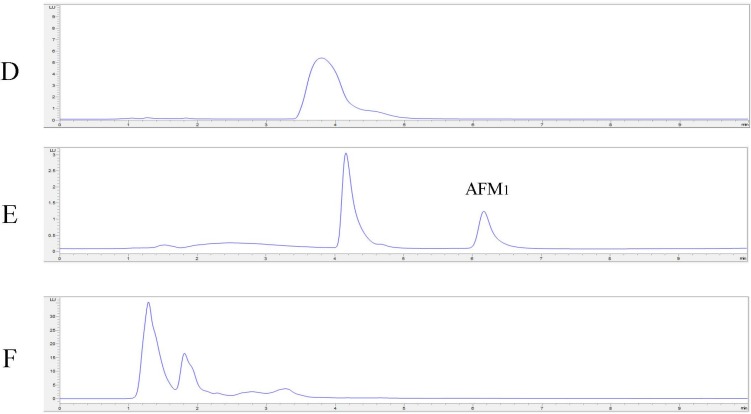
Examples of HPLC chromatograms of AFB_1_ (**A**—negative control, **B**—positive control, **C**—sample after degradation by Lac2) and AFM_1_ (**D**—negative control, **E**—positive control, **F**—sample after degradation by Lac2).

Degradation results are shown in Figure 2, while examples of HPLC chromatograms of AFB_1_ and AFM_1_ degradation are shown in Figure 3. Direct oxidation of AFB_1_ by means of Lac2 alone accounted for 23% degradation. The addition of a redox mediator resulted in a very effective degradation of the toxin. The lowest concentrations of ABTS and AS (1 mM) were able to double the degradation percentage compared to Lac2 alone (45% and 42%, respectively) while, in the case of SA, the presence of mediator at 1 mM lowered the degradation percentage (13%). Absolute values of aflatoxin concentrations are shown in Table 3.

At 10 mM each mediator further enhanced AFB_1_ degradation which reached 90% for AS, 81% for ABTS and 72% for SA. With regards to AFM_1_, Lac2 proved to completely degrade the toxin with all mediators added at 10 mM since after the reaction no AFM_1_ peak was detected by HPLC analysis.

## 3. Discussion

In this work we purified and identified a Lac2 isoform from a strain of *P. pulmonarius* and evaluated the ability of the pure enzyme to degrade AFB_1_ and AFM_1_ either by direct or mediated oxidation with three different redox mediators, ABTS, AS, and SA.

Mycotoxin degradation by fungi and bacteria is a widely investigated topic, especially in the last 10–15 years [21,22,23], but a deep understanding of which enzyme is directly responsible for mycotoxin degradation and the mechanism involved, is still lacking. Many bioremediation applications exploit the fungus as a whole-cell biocatalyst, or its secretome, thus involving the concerted activity of several enzymatic systems, including laccases, extracellular peroxidases (Lignin peroxidase-LiP, EC 1.11.1.14, manganese peroxidase-MnP, EC 1.11.1.13, and versatile peroxidase-VP, EC 1.11.1.16) and oxidases that generate the H_2_O_2_ needed for peroxidase activity (tyrosinase-EC 1.14.18.1 and aryl-alcohol oxidase-EC 1.1.3.7). Furthermore, low molecular weight compounds that act as mediators might be present in the culture media. This limits the discrimination between the direct action of the enzyme and the mediated one, which is a crucial point to develop industrial or biotechnological applications. Even commercial fungal preparations may contain contaminant proteins: Margot and colleagues [24] recently verified that the most used commercial laccase from Sigma (Milan, Italy, Ref. 38429) actually contains a mixture of different proteins, from 17 to ~80 kDa, and different LC isoforms.

Although aflatoxin degradation by fungal laccase enzymes has already been reported [15,25,26], to date no direct and unambiguous correlation between laccase and aflatoxin degradation has been described, since cultured filtrates or LC commercial preparation were tested in the degradation assays.

In our study the LC isozyme responsible for aflatoxins degradation was identified as Lac2. This isozyme has been extensively biochemically characterized in a previous work by Zucca and colleagues [27]. They reported a copper content of 3.8 cupric ions per protein molecule and a sugar content of 6.7% ± 0.3% (expressed as glucose equivalents), measured the enzyme activity at different pH values, and its stability at different temperatures.

Lac2 production was induced by low molecular weight compounds; among various putative inducers, ferulic acid proved to be by far the most effective [27]; according to this, the induced sample in zymography showed one band with a much more intense activity than the related NI sample. In order to remove contaminants and to purify laccase, a preparative chromatography was performed. As expected, this procedure increased the activity of bands detected in the NI and I samples (Figure 1, panel A) and resulted in a laccase band with an apparent molecular weight close to 35 kDa, as determined by the comparison with the lanes stained with Coomassie (Figure 1, panel B and C). Similar results were described by Diaz [28], who detected four laccase isoenzymes with molecular weights of 65, 47, 38, and 29 kDa.

The predicted molecular weight of Lac2 is 56.6 kDa, in agreement with the previous estimation by SDS PAGE and RP-HPLC–electrospray ionization-MS which assigned Lac2 a molecular weight of 55–61 kDa [27]. However, in this study Lac2 apparent molecular weight, estimated by SDS PAGE, was approximately 35 kDa; a possible explanation of this divergence is that under non-reducing conditions LC shows increased mobility and a lower apparent molecular weight due to the extensive glycosylation, as measured for this LC isozyme by Zucca et al. [27]. Moreover, glycosylation has been reported to be responsible for unconventional electrophoretic behavior under non-reducing or native conditions [29] and to influence SDS/protein interaction; it may facilitate LC migration through the gel net, making the LC external structure more flexible and elastic.

The limited reactiveness of Lac2 alone towards AFB_1_ might be explained by the high electrochemical oxidation potential, high ionization potential, or steric hindrance that prevents the substrate from being oxidized or enter the active site of LC [30]. Those limitations can be overcome by the use of redox mediators, which are effective LC substrates and, in turn, oxidize recalcitrant compounds.

Effective degradation of aflatoxins proceeds via LMS, both with ABTS and natural mediators, despite their proposed different mode of action. ABTS mediation is reported to occur via an electron transfer (ET) route, while phenoxy radicals mediate via hydrogen atom abstraction (HAT), at least when working at acidic or neutral pH values; only under alkaline conditions, where the mediators are in their anionic form, the HAT mechanism turns into an ET one [8,9,31].

ABTS was the first used artificial mediator [32]. It is soluble in water and, upon one-electron oxidation, produces a stable radical cation with a high absorbance at 420 nm. Thanks to these features ABTS has been successively used as an oxidation mediator towards polycyclic aromatic hydrocarbons, in organic synthesis, and in the treatment of textile wastewater [33,34,35]. ABTS is a model compound for LMS and an efficient mediator for AFB_1_ and AFM_1_ (81% and 100% degradation, respectively). However, it is a synthetic mediator and, as such, its high cost and concerns related to its potential toxicity have restricted industrial implementation of this LMS even in non-food applications, and raised the need for safe, cost-effective, and readily available mediators.

With this aim, we investigated the role of two naturally-occurring compounds, AS and SA, which are both 2,6-dimethoxy-substituted phenols derived from syringyl lignin units. They were described as the fastest and most efficient laccase mediators for the degradation of industrial dyes, sulfonamide antibiotics, and for the removal of lignin from paper pulps [36,37,38]. In addition to being natural compounds, they are inexpensive, safe, and can be as effective as the synthetic ones.

Both AS and SA were demonstrated to be effective mediators for AFB_1_ degradation at 10 mM, although SA was less efficient than AS and ineffective at 1 mM. Most probably, the SA-derived radical undergoes an internal redox reaction leading to syringic acid and, therefore, wasting a significant fraction of the reactive radical. Such a decrease of the reactive radical did not occur in the case of AS, explaining the noticeable effectiveness of the compound.

Methoxy substitutions in syringyl-type compounds decrease the redox potential and increase electron density at the phenoxy group. Those compounds are readily oxidized by LC and generate relatively stable radicals since the substitutions in the phenol ring impose steric hindrances for the polymerization via radical coupling [9,39]. The substituent in the para position also influences the phenoxy radical stability, since electron donor groups at the para-position stabilize the phenoxy radicals, while electron-withdrawing substituents lead to a decreased radical stability [40]. Accordingly, AS harbors the weakest electron acceptor group and generates a more stable radical than SA.

With respect to AFM_1_, Lac2 was able to degrade it completely with all mediators tested at 10 mM with no differences emerging among ABTS, AS, and SA. The decontamination of AFM_1_ in buffered solution, in model and real food matrices, was previously investigated using lactic acid bacteria [41]. Nevertheless, the decontamination was a result of a reversible binding to the carbohydrates and peptidoglycan of the bacterial cell wall surface and not a biological degradation. To our knowledge, the present study reports for the first time the effective degradation of AFB_1_ and AFM_1_ by means of Lac2 from *P. pulmonarius*.

The degradation products of aflatoxins have not been identified yet. Depending on the degrading agent, aflatoxins can be degraded by several mechanisms, such as epoxidation, hydroxylation, dehydrogenation, and reduction. A wide range of putative potential reaction products obtained accounts for the difficulties in the development of sensitive identification methods, as well as for the limited data on their toxicological characterization. Considering the degradation products deriving from laccase treatment, only a study conducted by Alberts and colleagues [15] reported a reduced mutagenicity (using Ames test) of the degradation products of AFB_1_.

According to our results we hypothesize that LMS acts on the lactone ring, which is responsible for fluorescence properties: cleavage of the lactone ring results in a non-fluorescent compound that has greatly reduced biological activity [42,43]. However, the ring cleavage of lactones is a hydrolysis, rather than an oxidation. Therefore, as laccases cannot catalyze lactone hydrolysis, fluorescence quenching should be the consequence of a deeper modification of the coumarin-like core of the toxins, which is responsible for their fluorescence. In fact, oxidative demethoxylations of simple aromatics by means of fungal laccases are well known [44,45]; methanol is released and the aromatic ring is changed into its quinonoid counterpart, which rearranges with the result of an irreversible and deep degradation. Hypothesizing that this mechanism is also valid for substituted aromatics, like aflatoxins, the reversibility of the reaction is unlikely.

This forecast is confirmed by our observations reported here. Additionally, as expected, the use of redox mediators strongly enhances the degrading ability of Lac2.

Laccase has been increasingly applied in food industry in the last 30 years. The demonstrated biodegrading activity towards mycotoxins, the green catalysis, and the use of natural mediators support a potential and feasible application in food and feed. A mandatory requisite for feed application is that products of mycotoxin degradation have to be stable and non-toxic. The development of these applications has to overcome the cost of production of the enzyme, the optimization of the degradation reaction, as well as the gap of knowledge related to the degradation products and their toxicity as required by the EU commission [46].

*Pleurotus* spp. ligninolytic system is a source of biotechnologically important enzymes which play an essential role in green bioremediation; so far, *P. pulmonarius* LCs have been extensively used in the removal of industrial dyes and the treatment of lignocellulosic waste.

This is the first time that *P. pulmonarius* Lac2 was unambiguously identified as capable of degrading AFB_1_ and AFM_1_ in the presence of natural redox mediators. Although further studies are needed to optimize the degradation assay, this study clearly illustrates the potentiality of Lac2 for its use as a biotransformation agent.

## 4. Materials and Methods

### 4.1. Organism, Culture Conditions, LC Induction, and Production

The *Pleurotus pulmonarius* strain ACR-16 of Cattedra di Chimica Biologica Collection, department of Biomedical Sciences, University of Cagliari (), syn. *P. sajor-caju*, [20], was maintained as ITEM17144 in the Agri-Food Toxigenic Fungi Culture Collection of the Institute of Sciences of Food Production, CNR, Bari [47]. ITEM17144 was routinely grown on malt extract agar plates (MEA, Oxoid) at 25 °C.

For LC production, ITEM 17144 was grown in liquid medium (2% *w*/*v* malt extract, 0.5% *w*/*v* yeast extract, 10 mM of potassium phosphate buffer pH 6, 0.1 mM of CuSO_4_) supplemented with 10 mM ferulic acid as a laccase inductor for 24 days, in darkness at 25 °C, in static conditions (relative humidity 70%).

### 4.2. Chemicals and Reagents and Standards Preparation

Chemicals for gel electrophoresis including Bio-safe Coomassie stain and Bradford reagent were supplied by Bio-Rad Laboratories (BioRad, Milan, Italy).

Acetonitrile (ACN) (LC–MS grade), formic acid, acetic acid, ammonium bicarbonate, trizma^TM^ base, tween 20, hydrochloric acid, trifluoroacetic acid (TFA), iodoacetamide (IAA), dithiothreitol (DTT), 2-azino-di-[3-ethylbenzo-thiazolin-sulphonate] (ABTS), acetosyringone (AS), syringaldehyde (SA), AFB_1_ and AFM_1_ standards (purity >99%) were obtained from Sigma-Aldrich (Milan, Italy). Trypsin (proteomic grade) was purchased from Promega (Milan, Italy). Regenerate cellulose syringe filters, 0.2 µm (size 4 mm) were obtained from Sartorius Italy S.r.l. (Muggiò, Italy)

Mycotoxin stock solution of AFB_1_ (10 µg/mL) was prepared by dissolving solid commercial toxins in toluene:ACN (9:1, *v*/*v*) (HPLC grade). The exact concentration of aflatoxin B_1_ was determined according to Association of Official Analytical Chemists (AOAC) Official Method 971.22 [48]. Aliquots of the stock solution were transferred to 4 mL amber silanized glass vials and evaporated to dryness under a stream of nitrogen at 50 °C. The residue was dissolved with water:methanol (60:40, *v*/*v*) to obtain calibrant standard solutions at 0.4, 1.2, 2.0, 4.0, 5.0, and 10.0 ng/mL. Standard solutions were stored at −20 °C and warmed to room temperature before use.

Mycotoxin stock solution of AFM_1_ (10 µg/mL) was prepared by dissolving solid commercial toxins in ACN (HPLC grade). The exact concentration of standard aflatoxin solution was determined according to AOAC official method 2000.08 [49]. Aliquots of the stock solution were transferred to 4 mL amber glass vials and evaporated to dryness under a stream of nitrogen at 50 °C. The residue was dissolved with water:ACN (75:25, *v*/*v*) to obtain calibrant standard solutions at 1.0, 2.5, 5.0, 7.5, and 10.0 ng/mL. Standard solutions were stored at −20 °C and warmed to room temperature before use.

### 4.3. LC Purification

LC purification was performed according to Zucca et al. [27] with slight modifications. Briefly, after 24 days of incubation, the culture medium was collected, diafiltered, and concentrated in 50 mM potassium phosphate buffer and 50 mM 6-aminohexanoic acid (protease inhibitor) using a Vivaflow 200 apparatus (Vivascience AG, Hannover, Germany) equipped with a Hydrosart membrane module (nominal MW cut-off 10,000 Da) and a Masterflex L/S system pump (Cole-Parmer, Vernon Hills, IL, USA) at 4 °C. The enzyme solution was added with NaCl to a final concentration of 0.25 M and gently stirred with freshly prepared calcium phosphate gel at 4 °C for 30 min. The slurry was centrifuged at 8000× *g* for 30 min, the supernatant recovered and diafiltered as previously described.

The resulting solution was adjusted to 0.2 M NaCl and loaded onto a DEAE-cellulose column (15 cm × 5 cm), which was pre-equilibrated with 50 mM potassium phosphate buffer pH 6 and 0.2 M NaCl. Bounded brown pigments were separated from unbound LC, which was eluted with the same buffer and desalted by dialysis against 50 mM potassium phosphate buffer prior to a second ion exchange chromatography.

Desalted LC fractions were loaded onto a Hiprep 16/10 DEAE FF assembled on an Akta Prime FPLC (Amersham Bioscience, Milan, Italy) equipped with a UV detector for protein absorbance monitoring at 280 nm. Column equilibration was performed with 50 mM potassium phosphate buffer pH 6 at a constant flow of 5 mL/min. Unbound proteins were washed out while LC was eluted with a linear gradient of 50 mM potassium phosphate buffer pH 6 containing 0.5 M NaCl in 40 min.

LC-rich fractions were pooled, concentrated, dialyzed, separated by size exclusion chromatography with a HiLoad 16/60 Superdex 75 column (GE Healthcare, Milan, Italy) assembled on the Akta Prime FPLC; equilibration and run were performed with 50 mM potassium phosphate buffer pH 6 at constant flow of 0.4 mL/min. Proteins were eluted and stored at −20 °C until use.

Protein content was determined using the Coomassie Brilliant Blue G250 method [50], the standard curve was performed using bovine serum albumin (BSA, 1–0.025 mg·mL^−1^).

### 4.4. LC Spectrophotometric Activity Assay

Laccase activity was photometrically measured (Ultraspec 3100pro, Amersham Pharmacia Biotech Italia, Cologno Monzese, Italy). The reaction was performed in 100 mM sodium acetate pH 4.5, 2 mM ABTS and an appropriate amount of enzyme solution in a final volume of 1 mL. The oxidation of ABTS was determined after 10 min by photometric assay at 420 nm (ε_420_ = 36,000 M^−1^·cm^−1^). One unit was defined as the amount of enzyme which oxidized 1 μmol of substrate per min [51].

### 4.5. Zymography 

The activities of the crude extract, both in presence (Induced, I) and absence (Not Induced, NI) of ferulic acid, as well as DEAE cellulose fractions were detected by zymograms, as previously reported [52].

Amounts of 5 µg (on average) both for I sample and the DEAE cellulose fraction, and 2 µg of NI samples, were dissolved in denaturant, non-reducing sample buffer (62.5 mM Tris-HCl pH 6.8, 25% glycerol, 2% SDS, and 0.01% Bromophenol Blue); all samples were loaded on two sodium dodecyl sulfate-polyacrylamide gels electrophoresis (SDS-PAGE, 12% T, 3% C), performed according to Laemmli [53]. The unstained molecular weight marker M12 (2.5–200 KDa, Life Technology, Waltham, MA, USA) was used as the reference. Electrophoretic separation was performed in a Miniprotean System (Biorad, Segrate, Italy) filled with running buffer composed of 25 mM Tris and 0.19 M glycine at 100 V for 15 min and 150 V for 1 h.

After the run, the gel was divided into two segments: one segment was washed with distilled water (four washes of 15 min and one of 30 min) at room temperature in order to remove SDS and then incubated with a solution of 5 mM ABTS in 50 mM potassium phosphate buffer pH 6; LC activity was revealed within six min. The remaining segment was fixed with 40% ethanol, 10% acetic acid 50% H_2_O for 30 min and then stained with Bio-safe Coomassie stain (Bio-Rad), following the manufacturer’s instructions.

Destained gels were digitally acquired by an Image Scanner III (GE Healthcare, Pittsburgh, PA, USA). The experiment was performed in two replicates. The putative laccase band corresponding to the induced isoform, detected in the electrophoretic pattern of DEAE sample stained with Coomassie, was excised and analysed by mass spectrometry.

### 4.6. LC-MS/MS Analysis

The excised gel band shown in Figure 1 was cut into small pieces and placed in 1.5 mL vials for in-gel digestion, with trypsin chosen as the proteolytic enzyme. Protein digestion was accomplished according to the manufacturer’s instructions with slight modifications. Firstly, gel slices were destained by adding 200 μL of 100 mM NH_4_HCO in 50% ACN and kept at 37 °C for 45 min; this step was repeated until electrophoresis dye was removed. Gel slices were then dehydrated in 100 µL of ACN and dried in a Speed Vac. Then, 130 µL of 10 mM DTT solution (prepared in 25 mM NH_4_HCO_3_) were added to the batch and incubated in shaking conditions for 1 h at 57 °C. Thirty microliters of 55 mM IAA solution (prepared in 25 mM NH_4_HCO_3_) were added and the mixture was incubated in darkness for 30 min at room temperature. After incubation, 0.3 µg of trypsin were added to the batch and incubated under shaking conditions overnight at 37 °C. The sample was then incubated with 150 µL of MilliQ water for 10 min, with frequent vortex mixing. Liquid was then removed and saved in a new microcentrifuge tube (Sigma, Milan, Italy). The extraction of gel slices digest was performed by adding 50 µL of 50% can, 5% TFA, 45% H_2_O solution. The extract was incubated under shaking conditions at room temperature for 1 h and centrifuged to recover the supernatant fraction. The procedure was repeated twice. Both supernatants were collected, mixed, and added with the aliquot obtained by sample incubation with water, and evaporated in the speed-vac in order to concentrate the sample. The final pellet was re-suspended in 50 µL of H_2_O:ACN (90:10 + 0.1% of formic acid) and filtered through regenerate cellulose filters (0.22 µm) before injection into the HPLC-MS system.

For the HPLC-MS/MS analysis a system consisting of UHPLC pump coupled through an ESI interface with a dual pressure linear ion trap mass spectrometer VelosPro^TM^ (Thermo Scientific, San José, CA, USA) was used. Peptide separation was performed on an Aeris peptide 3.6 µm XB-C-18 analytical column (150 × 2.10 mm, 3.6 µm, 100 Å, Phenomenex (Torrance, CA, USA); the injection volume was 20 µL. The following linear elution gradient was used for the analytical separation: solvent B was varied from 5%–60% in 55 min; then was increased up to 90% in 1 min and this ratio was maintained constant for the following 15 min. The precent of B was suddenly decreased at 5% and kept stable for 15 min for column reconditioning. The two reserves used were: A = H_2_O + 0.1% formic acid and B = ACN + 0.1% formic acid; flow rate was set at 200 µL/min.

MS system was operated in Data Dependent^TM^ Acquisition mode (DDA) by selecting the option *N*th *order double play* mode. In particular, two events were set for this experiment: (1) full MS in the range 400–2000 *m*/*z*, four microscans; and (2) full MS/MS DDA of the 20 most abundant ions in the MS spectrum using a normalized collision energy at 35%. Dependent settings for the DDA were set as reported [54].

### 4.7. Bioinformatic Analysis

Raw data obtained from LC-MS/MS acquisitions were searched against a customized database (DB = approximately 12,900 entries) containing amino acid sequences referred to all *Pleurotus* spp., downloaded from the largest UniProt DB available online [55] Protein identification was performed by the commercial software Proteome Discoverer^TM^ based on Sequest™ (version 1.4, Thermo-Fisher-Scientific, San José, CA, USA, 2012) scoring algorithm. Software results were filtered post-acquisition by peptide mass deviation (300 ppm), by setting *n* = 3 as minimum number of peptides for protein identification and peptide confidence medium (meaning better than 5% of confidence level).

### 4.8. In Vitro Degradation of AFB_1_ and AFM_1_ with LC and Redox Mediators

Degradation assays were performed in 500 µL of reaction volume of 1 mM sodium acetate buffer pH 5 with 1 µg/mL of AFB_1_. 2.5 units of LC were added to each reaction.

Alternatively, ABTS, AS, or SA were independently tested as redox mediators at 1 mM and 10 mM.

With respect to AFM_1_, degradation assays were performed by incubating 0.05 µg/mL of AFM_1_, 2.5 units of LC and ABTS, AS, or SA as redox mediators at 10 mM. In control samples, the enzymatic solution was replaced by an equal volume of buffer. Reactions were incubated at 25 °C for three days in the dark. Each experiment was performed in triplicate.

### 4.9. Chemical Analyses

AFB_1_ analyses were performed with a HPLC Agilent 1260 Series (Agilent Technology, Santa Clara, CA, USA) with post column photochemical derivatization (UVE™, LCTech GmbH, Dorfen, Germany). The analytical column was a Luna PFP (150 × 4.6 mm, 3 μm) (Phenomenex, Torrance, CA, USA) preceded by a SecurityGuard™ (PFP, 4 × 3.0 mm, Phenomenex).

Samples containing AFB_1_ were filtered using RC 0.20 µm filters (Grace) and 100 μL of volume was injected into the HPLC apparatus with a full loop injection system. The fluorometric detector was set at wavelengths of 365 nm (excitation) and 435 nm (emission). The mobile phase consisted of a mixture of H_2_O:ACN (70:30, *v*/*v*) and the flow rate was 1.0 mL/min. The temperature of the column was maintained at 40 °C. AFB_1_ was quantified by measuring peak areas at the retention time of aflatoxin standard solutions (Sigma-Aldrich, Milan, Italy) and comparing these areas with the relevant calibration curve at 0.4–10.0 ng/mL. With this mobile phase, the retention time was about 14.5 min. The limit of quantification (LOQ) was 0.4 ng/mL, while the LOD of the method was 0.2 ng/mL based on a signal to noise ratio of 3:1. 

AFM_1_ analyses were performed with a HPLC Agilent 1260 Series with a fluorometric detector (Santa Clara, CA, USA). The column used was a Zorbax SB-C18 (150 × 4.6 mm i.d., 5 μm Agilent, (Santa Clara, CA, USA) with a security guard (4 × 3.0 mm).

AFM_1_ levels in samples were determined by HPLC/FLD method. The solutions were filtered using RC 0.20 µm filters (Grace, Taipei, China); 50 μL were injected into the HPLC apparatus with a full loop injection system. The fluorometric detector was set at wavelengths of 365 nm (excitation) and 450 nm (emission). The mobile phase consisted of a mixture of H_2_O:ACN (75:25, *v*/*v*) and the flow rate was 1 mL/min. The temperature of the column was maintained at 30 °C. AFM_1_ was quantified by measuring peak areas at the retention time of aflatoxin standard solutions and comparing these areas with the relevant calibration curve at 1.0–10.0 ng/mL. With this mobile phase, the retention time of AFM_1_ was about 6 min. The LOQ was 1.0 ng/mL, while the limit of detection (LOD) of the method was 0.12 ng/mL, based on a signal to noise ratio of 3:1.

When needed, controls and samples were diluted to fit the calibration ranges of the corresponding HPLC methods. Degradation percentages were calculated as follows:

(1) $$\%\;aflatoxin\;degradation=\frac{aflatoxin_{sample}}{aflatoxin_{control}}\times 100$$

## Figures and Tables

**Table 1:** Summary of Lac2 purification from *P. pulmonarius* culture filtrate.

Purification Step	Total Volume (mL)	Total Activity (EU)	Total Protein (mg)	Specific Activity (U/mg)	Purification Fold
Crude extract	4000	17,120	2800.00	6	1
Ca-phosphate gel	500	33,400	95.00	351	59
DEAE cellulose	50	23,785	11.50	2068	344
DEAE FF	10	3837	1.20	3224	538
Superdex	14	2053	0.19	10,920	1820

**Table 2:** Overview of protein assignments referred to the analysis of the excised band obtained by the Sequest scoring algorithm interrogating a customized *Pleurotus* database imported by UniProt.

Assigned Protein	Accession Number	Protein Coverage	No. of Identified Peptides	Sequences	Confidence Level	*m*/*z* (Da)
**Laccase 2** *Pleurotus pulmonarius*	Q2VT18	21.24%	7	YSFVLTADQTPDNYWIR	High	1045.07686
				YAGGPTSPLAVINVESTKR	High	980.65433
				SAGSTTYNFDTPAR	High	744.42450
				GDNFQLNVVNQLSDTTMLK	High	1069.20405
**Laccase 4** *Pleurotus sajor-caju*	Q7Z8S3			SAGSTTYNFDTPARR	High	822.55662
				ANPNLGSTGFAGGINSAILR	High	644.12372
				SVPITGPTPATASIPGVLVQGNK	High	735.54871
				GDNFQLNVVNQLSDTTMLK	High	718.38048

**Table 3:** Absolute values of aflatoxin concentrations (ng/mL) after LC treatment and statistical analysis of aflatoxin degradation by Lac2 and redox mediators.

Sample	AFB_1_		AFM_1_
	med 1 mM	med 10 mM	med 10 mM
Positive control	923 ± 33 *	923 ± 33 *	53 ± 7 *
No med	710 ± 27 *	710 ± 27 *	n.t.
ABTS	508 ± 46 *	175 ± 5 *	0 ± 0 *
AS	535 ± 9 *	92 ± 27 *	0 ± 0 *
SA	803 ± 120	258 ± 16 *	0 ± 0 *

n.t. = not tested; Comparisons between controls and treated samples were performed using a *t*-test. A *p* value < 0.001 was considered statistically significant (*).

## Abbreviations

ABTS	[2,2′-azino-bis-(3-ethylbenzothiazoline-6-sulfonic acid)]
CAN	Acetonitrile
AFB_1_	aflatoxin B_1_
AFM_1_	aflatoxin M_1_
AS	Acetosyringone
DEAE	diethylamino ethyl
ET	electron transfer
EU	enzymatic unit
FLD	fluorescent detector
HAT	hydrogen atom abstraction
HBT	Hydoxybenzotriazole
Lac2	laccase 2
LC	laccase
LMS	laccase mediator system
LOD	limit of detection
LOQ	limit of quantification
SA	Syrinagaldehyde
TEMPO	2,2,6,6-tetramethylpiperidine-*N*-oxyl
